# Pilot Study of Sodium-Glucose Cotransporter 2 Inhibitor Empagliflozin Shows Reduced Intrarenal Complement Activation in Patients With Diabetes and CKD

**DOI:** 10.1016/j.ekir.2024.11.014

**Published:** 2024-11-18

**Authors:** Mia Jensen, Steffen F. Nielsen, Steffen Thiel, Søren W.K. Hansen, Yaseelan Palarasah, Per Svenningsen, Jesper N. Bech, Frank H. Mose, Boye L. Jensen

**Affiliations:** 1 Unit of Cardiovascular and Renal Research, Department of Molecular Medicine, University of Southern Denmark, Odense, Denmark; 2 University Clinic in Nephrology and Hypertension, Gødstrup Hospital, Herning, Denmark; 3 Department of Clinical Medicine, Aarhus University, Aarhus, Denmark; 4 Department of Biomedicine, Aarhus University, Aarhus, Denmark; 5 Unit of Cancer and Inflammation Research, Department of Molecular Medicine, University of Southern Denmark, Odense, Denmark

**Keywords:** kidney injury, lectin cascade, type 2 diabetes mellitus

## Introduction

Sodium-glucose cotransporter 2 inhibitors (SGLT-2i’s) improve kidney and cardiovascular outcomes in patients with diabetic nephropathy beyond antihypertensive and glucose-lowering effects.^1^ In patients with albuminuria, complement precursors are aberrantly filtered and subsequently activated in tubular fluid.^2^^,^^3^ Patients with diabetes and albuminuria are prone to complement binding to metabolically stressed and hyperactive SGLT-2–expressing tubular cells. Pattern-recognition molecules in the lectin pathway of complement, including MBL, collectin kidney 1 (CL-K1), and collectin liver 1 are associated with diabetic nephropathy.^4^ CL-K1 is synthesized in the proximal tubules and contributes to kidney injury in mice.^5^^,^^6^ In rats with experimental chronic kidney disease (CKD), treatment with SGLT-2i lowers the expression of complement system components C1qa and -c in the kidneys.^7^ In plasma from patients with diabetes and albuminuria, collectin concentrations are not different after SGLT-2i treatment.^8^ We hypothesized that SGLT-2i’s attenuate local, intratubular, complement activation in patients with diabetes and CKD. To that end, we explored the effect of the SGLT-2i, empagliflozin, on collectins and complement system activation and deposition in plasma, urine, and tubular membranes by urine microvesicles from patients with type 2 diabetes mellitus (DM) with and without CKD compared with patients with nondiabetic CKD. Samples were from double-blinded, randomized, placebo-controlled, exploratory, crossover trials designed to study the complement system and acute changes in renal blood flow and glomerular filtration rate (GFR) in response to SGLT-2i–“SiRENA”^9^: patients with type 2 DM (DM, *n* = 16), patients with type 2 DM and CKD (DM-CKD, *n* = 17), and with nondiabetic CKD (CKD, *n* = 16) (Supplementary Figure S1). Patients were randomized to receive empagliflozin 10 mg/d or placebo. After 4 weeks, patients were crossed over to the opposite treatment for 4 weeks after a minimum of 2 weeks of washout.

## Results

At baseline, GFR was lower, and albuminuria was higher in CKD groups with/without diabetes compared to patients with DM only. HbA1c was higher in patients with diabetes compared with patients with CKD only (Supplementary Table 1). Empagliflozin lowered blood pressure in the DM and DM-CKD groups and urine albumin-to-creatinine ratio in the DM-CKD group compared with placebo (Table 1). GFR decreased modestly but significantly in all groups following empagliflozin, but no change was observed in HbA1c (Table 1). Analysis of treatment periods for placebo and empagliflozin showed no significant carryover effects (Supplementary Table S3–S5). There were no significant differences in plasma concentration of collectins and MASP-2 between placebo and empagliflozin treatment (Supplementary Table S1). Of note, collectins, CL-K1 and collectin liver 1 were higher in plasma from patients with DM than both CKD groups. There was a direct relation between plasma concentrations of CL-K1 and collectin liver 1 in both treatment periods in all 3 groups (Supplementary Figure S2). It could be speculated that in patients with CKD, collectins are lost in urine or degraded at a higher rate. There were no significant differences in plasma concentration of C3a, C5a, and sC5b-9 between placebo and empagliflozin treatment (Supplementary Table S2). C3dg increased significantly (18.1%, *P* < 0.03) in response to empagliflozin in the DM-CKD group compared with placebo (Supplementary Table S2) with no significant differences in other groups. Taken together, empagliflozin did not lower collectins or complement activation in plasma in the patient groups.

**Table 1:** Clinical data of included patients

Clinical data	DM (*n* = 16)		CKD (*n* = 16)		DM-CKD (*n* = 17)	
	Placebo	Empagliflozin	Placebo	Empagliflozin	Placebo	Empagliflozin
Blood pressure (mmHg)						
Systolic	135 ± 9	130 ± 11^a^	125 ± 11	121 ± 13	140 ± 15	132 ± 12^b,c^
Diastolic	79 ± 8	76 ± 10^a^	79 ± 9	77 ± 9	79 ± 9	76 ± 7^a^
HbA1c (mmol/mol)	53 ± 7	54 ± 8^c^	36 ± 2	36 ± 2	55 ± 8	55 ± 7^c^
UACR (mg/g)	18 [12–99]	20 [9–88]	87 [17–1000]	76 [32–1093]^c^	201 [62–1130]	164 [44–719]^a.c^
eGFR (ml/min per 1.73 m^2^)	89 ± 20	78 ± 19^cd^	36 ± 12	30 ± 11^a^	42 ± 10	39 ± 12^a^

CKD, chronic kidney disease; DM, diabetes mellitus; eGFR, estimated glomerular filtration rate; IQR, interquartile range; UACR, urine albumin-to-creatinine ratio.

Data are presented as mean ± SD or median [IQR].

Significant difference intragroup between treatments: ^a^*P* < 0.05, ^b^*P* < 0.01, ^d^*P* < 0.0001.

^c^Significant difference between the groups.

**Figure 1:**
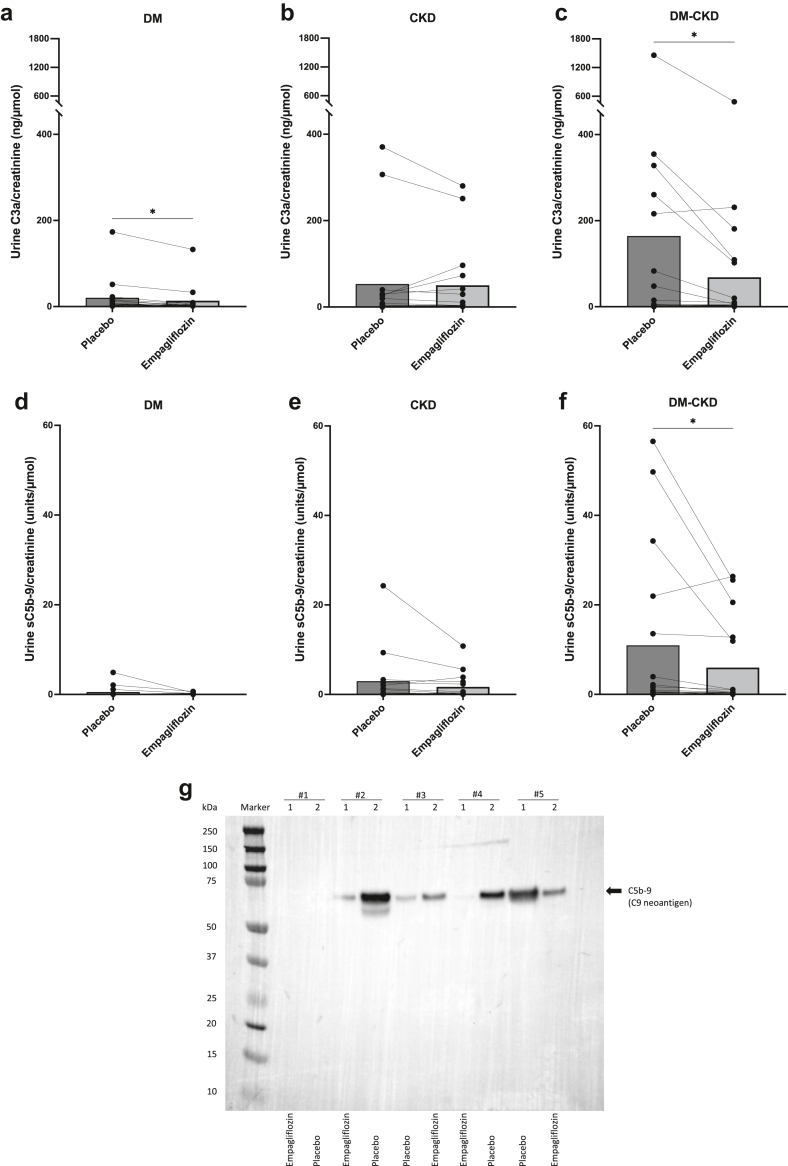
Concentration of complement fragment C3a and the membrane attack complex (sC5b-9 neoepitope) in crude spot urine samples as creatinine ratio. Each patient is represented by connected dots, and columns depict mean values. Determination of C3a concentration showed a significant decrease following empagliflozin in patients with (a) DM and (c) DM-CKD, but no change was observed in C3a levels in patients with CKD (b). Soluble C5b-9-to-creatinine ratio decreased significantly following empagliflozin in (f) DM-CKD compared to placebo, but no change was observed in sC5b-9 levels in (d) DM or (e) CKD. Data were evaluated by using paired *t* test, ∗*P* < 0.05. (g) Immunoblotting for C5b-9-neoepitope after SDS-PAGE separation of proteins in urine extracellular vesicles isolated from patients normalized for urine creatinine. Samples for extracellular vesicle isolation and immunoblotting were selected based on enzyme-linked immunosorbent assays determination of C5b-9 as follows: 1 DM-CKD participant (#1) displayed a low level of C5b-9 in urine and 4 DM-CKD participants (#2–5) with high levels. Each participant had urine collected after the first and second intervention period with empagliflozin or placebo treatment. The expected migration pattern or molecular size of C5b-9 is ∼61 kDa. CKD, chronic kidney disease; DM, diabetes mellitus.

CL-K1 and collectin liver 1 concentrations in crude urine without or with *ex vivo* concentration did not surpass detection limit. In contrast, complement activation products were detectable in urine samples (Figure 1 and Supplementary Figure S3). C3a was detected in 96% of all samples and decreased significantly in response to empagliflozin in the DM group (32%, *P* < 0.0110) (Figure 1a) and DM-CKD group (58%, *P* < 0.0129) (Figure 1c), with no change in the CKD group (Figure 1b). The stable C3 activation product, C3dg, was detected in 12.5% of all samples from DM and 28% of patients with CKD and 47% of DM-CKD (Supplementary Figure S3A–C). No significant differences were observed between treatments in C3dg. C5a was detected in a minority of samples in the DM group (19%) (Supplementary Figure S3D), 41% of patients in the CKD group, and 47% in the DM-CKD group (Supplementary Figure S3E and F), but with no significant changes in response to treatment. The membrane attack complex, sC5b-9, was detected in 44% of urine samples from the DM group (Figure 1d), 63% in CKD (Figure 1e), and 79% in DM-CKD (Figure 1f). sC5b-9 decreased significantly in response to empagliflozin in the DM-CKD group (45.4%, *P* < 0.02), whereas no changes were observed in the DM or the CKD groups. Urine albumin concentration correlated significantly and strongly with sC5b-9, C3a, C5a, and C3dg in the DM-CKD, and less in the CKD and DM groups (Supplementary Table S6). Extracellular vesicles were isolated from urine after treatments and selected from 4 patients with DM-CKD with high and changed levels of sC5b-9 after SGLT-2i, and from 1 patient (control) with low and unchanged levels. Immunoblotting for C5b-9 corroborated the findings from enzyme-linked immunosorbent assays at the level of apical membranes, such that those displaying a high concentration had detectable membrane deposition and responded to empagliflozin with lower levels in 3 out of 4 samples (Figure 1g).

## Discussion

The study demonstrates that the SGLT-2i, empagliflozin lowers albuminuria, C3a, and the membrane attack complex in urine from patients with DM and CKD but does not affect plasma levels. Patients with type 2 DM display higher levels of circulating collectins but not MASP2. Based on these results and other results,^8^ it is unlikely that SGLT-2i’s as class exert systemic antiinflammatory effects by reducing circulating levels of collectins or plasma complement activation. Rather, empagliflozin may lower complement activation by a local intrarenal event detectable in urine. Complement products in urine related directly to albuminuria in line with previous studies.^2^^,^S1^,^S2 The prime novel observation was that, empagliflozin lowers anaphylatoxin C3a in urine from patients with DM and DM-CKD and the membrane attack complex (C5b-9) in the DM-CKD group only. Thus, diabetes, but not albuminuria alone, appears necessary to uncover the full inhibitory effect of SGLT-2i’s on renal complement activation, a notion that should be proven in larger studies. One interpretation would be that a combination of filtration barrier defect with aberrant presence of complement precursors in tubular fluid and concomitant proximal tubular metabolic stress by increased glucose load and amplified SGLT-2 activity and expressionS3 prompts local complement activation and deposition. The changes in urine were observed with similar levels in plasma and with a significant decrease in GFR. This supports complement activation and membrane deposition from the luminal side. This was corroborated by similar changes of C5b-9 in apical membranes. A decrease in both GFR and albuminuria could contribute to, but not fully account for, SGLT-2–mediated decrease in soluble and deposited C5b-9, because the effect on albumin excretion was modest (24%) compared to the reduction of more than 50% in the membrane attack complex (sC5b-9). It is believed that activation of the tubuloglomerular feedback system by SGLT-2i’s significantly reduces intraglomerular pressure, and thereby accounts for some of the renoprotection;^7^ whereas other findings show that macrophage activation in a rat model is attenuatedS4, which would be in line with the present findings on less anaphylatoxin C3a which is a powerful attractant for macrophages. The inhibitory effects of SGLT-2i’s on macrophage differentiation may account for cardiovascular protection.S5 Based on the present study, we conclude that SGLT-2i attenuates intrarenal complement activation and membrane deposition in diabetes.

## Disclosure

All the authors declared no competing interests.

## Supplementary Material

Supplementary File (PDF) **Supplementary Methods. Supplementary References. Figure S1.** Study design of the SiRENA trial. **Figure S2.** Correlation between collectin kidney 1 and collectin liver 1. **Figure S3.** Spot urine concentrations of C3dg and C5a. **Table S1.** Concentration of lectin pathway–associated molecules in plasma. **Table S2.** Concentration of complement activation products in plasma. **Table S3.** The difference between both evaluation periods in diabetes mellitus. **Table S4.** The difference between both evaluation periods in chronic kidney disease. **Table S5.** The difference between both evaluation periods in diabetes mellitus- chronic kidney disease. **Table S6.** Correlation between complement products and albu min in urine.

## Footnotes

Supplementary File (PDF)

**Supplementary Methods.**

**Supplementary References.**
**Figure S1.** Study design of the SiRENA trial. **Figure S2.** Correlation between collectin kidney 1 and collectin liver 1. **Figure S3.** Spot urine concentrations of C3dg and C5a. **Table S1.** Concentration of lectin pathway–associated molecules in plasma. **Table S2.** Concentration of complement activation products in plasma. **Table S3.** The difference between both evaluation periods in diabetes mellitus. **Table S4.** The difference between both evaluation periods in chronic kidney disease. **Table S5.** The difference between both evaluation periods in diabetes mellitus- chronic kidney disease. **Table S6.** Correlation between complement products and albumin in urine.
