# Supplementary material for: Pilot Study of Sodium-Glucose Cotransporter 2 Inhibitor Empagliflozin Shows Reduced Intrarenal Complement Activation in Patients With Diabetes and CKD
Source: Kidney Int Rep. 2024 Nov 18;10(2):591–5. doi: 10.1016/j.ekir.2024.11.014 (PMC11843100; doi:10.1016/j.ekir.2024.11.014)
Supplement: Supplementary File (PDF) — Supplementary Methods. Supplementary References. Figure S1. Study design of the SiRENA trial. Figure S2. Correlation between collectin kidney 1 and collectin liver 1. Figure S3. Spot urine concentrations of C3dg and C5a. Table S1. Concentration of lectin pathway–associated molecules in plasma. Table S2. Concentration of complement activation products in plasma. Table S3. The difference between both evaluation periods in diabetes mellitus. Table S4. The difference between both evaluation periods in chronic kidney disease. Table S5. The difference between both evaluation periods in diabetes mellitus- chronic kidney disease. Table S6. Correlation between complement products and albu min in urine. [file mmc1.pdf]

Supplementary Material

Pilot study of SGLT-2 inhibitor empagliflozin shows reduced intrarenal complement activation in patients with diabetes and chronic kidney disease

Mia Jensen[1], Steffen F. Nielsen[2,3], Steffen Thiel[4], Søren W.K. Hansen[5], Yaseelan Palarasah[5], Per Svenningsen[1], Jesper N. Bech[2,3], Frank H. Mose[2,3], & Boye L. Jensen[1]

Supplementary Methods

Study design and patients

The present study used samples from a clinical study designed to study renal blood flow, GFR, collectins, and complement activation changes in patients with diabetes and chronic kidney disease after administration of SGLT-2 inhibitors (SGLT2i) designated as SiRENA (S6). Three parallel groups were investigated in a randomized, double-blinded, placebo-controlled cross-over design (EudraCT 2019-004303-12, 2019-004447-80, and 2019-004467-50) that compared empagliflozin treatment with a matching placebo (Supplementary Fig. I). The studies included: 16 patients with type 2 diabetes mellitus (DM), designated as the DM group, 17 patients with DM and chronic kidney disease (CKD), defined as the DM-CKD group, and 16 patients with non-diabetic CKD, designated as the CKD group. Patients were eligible if they were at least 18 years of age. Inclusion criteria in the DM group was an HbA1c between 48-70 mmol/mol and an eGFR above 60 mL/min/1.73 m^2; in the DM-CKD group, an HbA1c between 48-70 mmol/mol and an eGFR between 20-60 mL/min/1.73 m^2; in the CKD, HbA1c below 48 mmol/mol and an eGFR between 20-60 mL/min/1.73 m^2. All included patients with diabetes were diagnosed with type 2 DM at least one year before inclusion and were on a stable dose of antidiabetic treatment at least three months before the start of the study drug. In all three studies, patients with anamnestic or clinical signs of significant liver disease or heart failure, as well as patients with type 1 DM, BMI >35 kg/m2, or patients with active cancer disease, previous kidney transplants, and polycystic kidney disease were excluded. Participants were recruited through newspaper advertisements, e-mails, or letters to participants of earlier trials and from the outpatient clinics at The Department of Internal Medicine, Nephrology and The Department of Internal Medicine, Endocrinology, Gødstrup Hospital, Denmark. The design of the three studies was identical. After a brief run-in period, the patients were randomized to receive empagliflozin 10 mg/day or matching placebo. After four weeks, the participants were crossed and assigned to the opposite treatment for another four weeks with a minimum of two weeks washout before treatment started. Plasma and spot urine samples were collected after both evaluation periods for all participants, and clinical parameters and complement factors were measured in the collected samples (Supplementary Fig. I). 2x1 mL of the spot urine was collected and saved for the measurement of complement products, and 40-50 mL were protease inhibited with one tablet of cOmplete™ Protease Inhibitor Cocktail (11697498001, Roche) for the purification of urinary extracellular vesicles (uEVs). 1x6 mL of blood was collected in K-EDTA tubes and centrifuged at 1000g for 15 minutes at 4 °C to obtain the plasma. All urine and plasma samples were saved at -80 °C until use. The studies were approved by the Scientific Ethics Committee of the Central Jutland Region (1-10-72-214-20, 1-10-72-339-20, 1-10-72-340-20) and the Danish Health and Medicine Authority (2020063197, 2021010055, 2022093892). The collection of samples was

performed in accordance with the Helsinki Declaration, the EU Directive on Good Clinical Practice (GCP), and International Conference of Harmonization (ICH-GCP) guidelines and all participants gave written and oral informed consent before inclusions.

Enzyme-Linked Immunosorbent Assays

In-house developed, published, sandwich ELISAs were used to measure the pattern recognition molecules collectin kidney 1 (CL-K1), collectin Liver 1 (CL-L1), mannan-binding lectin (MBL), the MBL associated serine protease 2 (MASP-2) and the complement split products C3a, C5a, C3dg, and the membrane attack complex (sC5b-9) in plasma and crude urine. The assays are described in detail in previous papers (S7-S15). In brief, all plates were coated in carbonate coating buffer (35 mM NaHCO3 and 15 mM Na2CO3, pH 9.6) with diluted primary antibody. CL-K1 was measured by in-house monoclonal mouse CL-K1 (5 µg/mL) and detected by in-house biotinylated CL-K1 16-25 (0.5 µg/mL) (S7). CL-L1 was measured by in-house monoclonal mouse CL-L1 (5 µg/mL) and detected by biotinylated in-house CL-L1 16-13 (0.5 µg/mL) (S8). MBL was measured by mouse monoclonal α-MBL HYB-131 (2.0 µg/mL, Statens Serum Institut) and detected by biotinylated mouse monoclonal α-MBL HYB-131 (1:500) (S9). MASP-2 was measured by rat monoclonal MASP-2 Human Clone 8B5 (4 µg/ml, Cat. #: HM2190B, Hycult Biotech) and detected by rat monoclonal 6G12 anti-MASP-2 (1.5 µg/ml, Cat. #: HM2191, Hycult Biotech) (S10). C3a and C5a are anaphylatoxins and are cleaved from complement components 3 (C3) and 5 (C5), essential for complement activation. C3a were measured by mouse monoclonal α-C3a (2.0 µg/mL, Cat. #: GAU 017-10, Bioporto) and detected by biotinylated mouse monoclonal α-C3a (1:2000, Cat. #: GAU 013-16, Bioporto) (S11). C5a was measured by mouse monoclonal anti-C5a (5 µg/mL, Cat. #: GAU 25-5, Bioporto) and detected by biotinylated rabbit polyclonal anti-C5a (1 µg/mL, Cat. #: PA5-35000, Invitrogen) (S11). C3dg is a stable activation end-product of C3. C3dg was measured by rat monoclonal α-C3dg 15-39-06 (2.0 µg/mL) and detected by biotinylated rabbit polyclonal α-C3dg (1:500, Cat. #: A0062, Agilent-DAKO) (S12, S13). The terminal product of the complement system is the membrane attack complex (C5b-9), which consists of C5b, C6, C7, C8, and multiple C9 molecules. C9 neoantigen was measured by mouse monoclonal α-C9 neoantigen WU13-15 (S14) (2.0 µg/mL, Cat. #: HM2264, Hycult Biotech) and detected by an antibody pool of biotinylated mouse monoclonal α-C9 (1:500 of α-C9 8-12-67 and α-C9 8-12-71, Bioporto) (S15). All plates were developed with streptavidin-conjugated horseradish peroxidase (Invitrogen) and 3.3´,5.5´-tetramentylbenzidine (TMB Ultra, Cat. no: 34028, Thermo Fisher Scientific or TMB-One, Cat. no: 4380, Kementec), except MASP-2 which was developed by europium-labeled streptavidin (PerkinElmer) and enhancement buffer. A Victor X5 multimode plate reader (Perkin Elmer) was used to read MASP-2 plate europium counts, and a Vmax microplate reader (Molecular Devices) at 450 nm was used to read all other plates.

Isolation of uEVs

Urine extracellular vesicles (uEVs) were isolated by polyethylene glycol (PEG) precipitation as previously described (S15, S16). Five mL of urine was thawed and centrifuged for 15 minutes at 5.000 g (Centrifuge 5910 R, Eppendorf) to remove cellular debris and cells. The supernatant was removed from all samples and transferred to a new tube with five mL of fresh PEG6000 (528877, Sigma-Aldrich) with 1 M NaCl. Samples were incubated overnight at 4 °C and were centrifuged for 15 min at 5.000 g (Centrifuge 5910 R, Eppendorf) at 4 °C. The supernatant was removed from all tubes, and the tubes dried upside down for 10 minutes. The pellet was re-suspended in 500 µL PBS. All samples were normalized to urine creatinine by dilution before immunoblotting.

Immunoblot analysis

The isolated uEVs were analyzed with immunoblotting by monoclonal antibodies directed against complement split products and uEV markers. Electrophoresis was performed using the NuPAGE system (Invitrogen) with 4-12% tris-bis gel and MES running buffer (Invitrogen). Samples were run under denaturing condition and for that purpose were mixed with NuPAGE lithium dodecyl sulfate (LDS) sample buffer and reducing agent and heated to 95°C for 5 min. Precision Plus Protein Dual Color Standard (Bio-Rad) was included as a molecular weight marker. Gels were blotted onto PVDF membranes and blocked with 2% non-fat skim milk (Sigma-Aldrich). The membranes next incubated with mouse monoclonal anti-C9 neoantigen WU13-15 (1:1500, HM2264, Hycult Biotech) diluted in 5% skim milk with tris buffered saline with 0.05% Tween20 (TBST), for 1 hour at room temperature or 4°C overnight. Horseradish peroxidase (HRP)-conjugated goat polyclonal anti-mouse (1:2000, P0447, Dako) diluted in 5% skim milk with TBST served as a secondary antibody. Proteins were visualized by ChemiDocXRS+ and image lab software version 6.1 (Bio-Rad laboratories).

Statistical power

The primary endpoint of the study was to explore the effect of empagliflozin on renal blood flow (RBF, EudraCT 2019-004303-12, 2019-004447-80, and 2019-004467-50), and because the analysis of the complement products was a secondary endpoint, no formal power calculation was performed for this sub-study. However, in our previous study of sC5b-9-associated C9 neoantigen (MAC complex) in urine from patients with type 1 diabetes mellitus with (n=15) and without nephropathy (n=12) in response to amiloride, we observed that sC5b-9 was detectable in 10 of 15 patients with nephropathy (S11). sC5b-9 was related to albuminuria and the sC5b-9-associated C9 neoantigen/creatinine ratio was significantly reduced in response to amiloride treatment (S11). Thus, we considered 15 patients to be sufficient to detect a relevant difference in an exploratory trial. Moreover, C3dg was detectable in at least one of two paired samples from 5 of 15 nephropathy patients, and in those patients,

C3dg/creatinine decreased significantly (S11). In most control subjects, urine C3dg/creatinine and sC5b-9-associated C9 neoantigen/creatinine ratios were below the detection range and no effect of amiloride was therefore observed.

Statistics

All datasets were analyzed using the D'Agostino & Pearson omnibus normality test for normal distribution. Normally distributed values were reported as mean ± standard deviation (SD), and non-normal distributed values were reported as median [interquartile range (IQR)]. Comparison between treatments was performed using paired parametric or non-parametric two-tailed t-tests. One-way ANOVA was performed to determine differences between the groups. Pearson's correlation was used to determine the correlation between two variables. All statistical analysis was performed using GraphPad Prism software version 9. $P<0.05$ was considered statistically significant.

Supplementary Figure S1

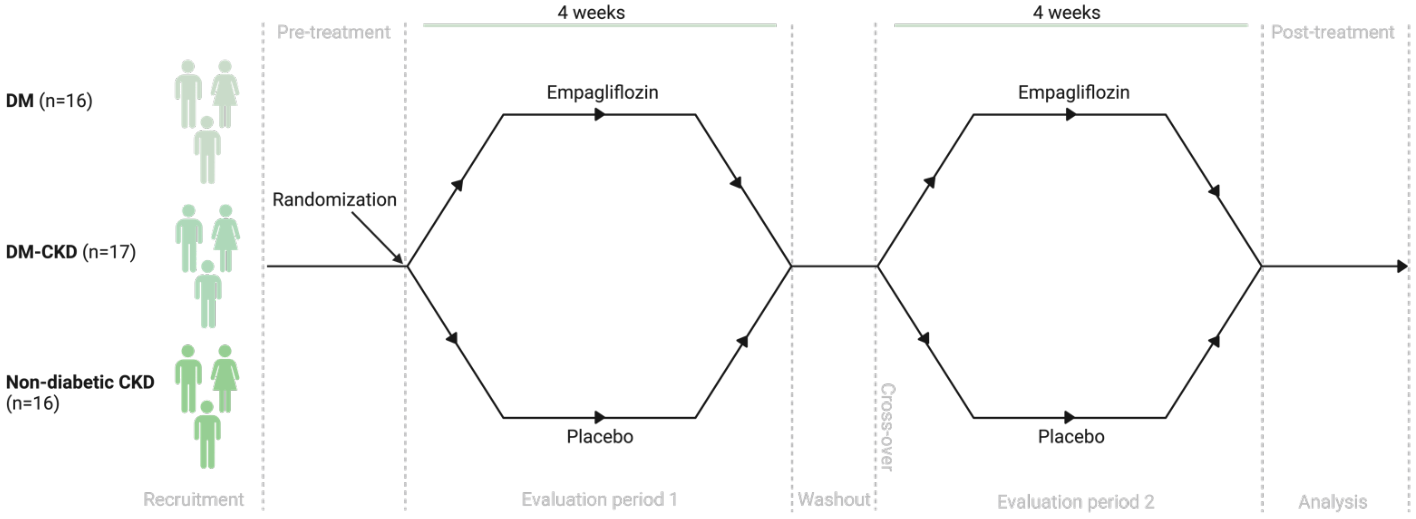

Figure S1: Study design of the SiRENA trial. A randomized, double-blind, placebo-controlled, cross-over study of patients treated with empagliflozin (10 mg/day) and placebo for 2x4 weeks with washout between treatments. A total of 49 patients with type 2 diabetes mellitus without (DM group, n=16) and with chronic kidney disease (DM-CKD group, n=17), and non-diabetic CKD were included in the study (CKD group, n=16). Created with Biorender.com

Supplementary Figure S2

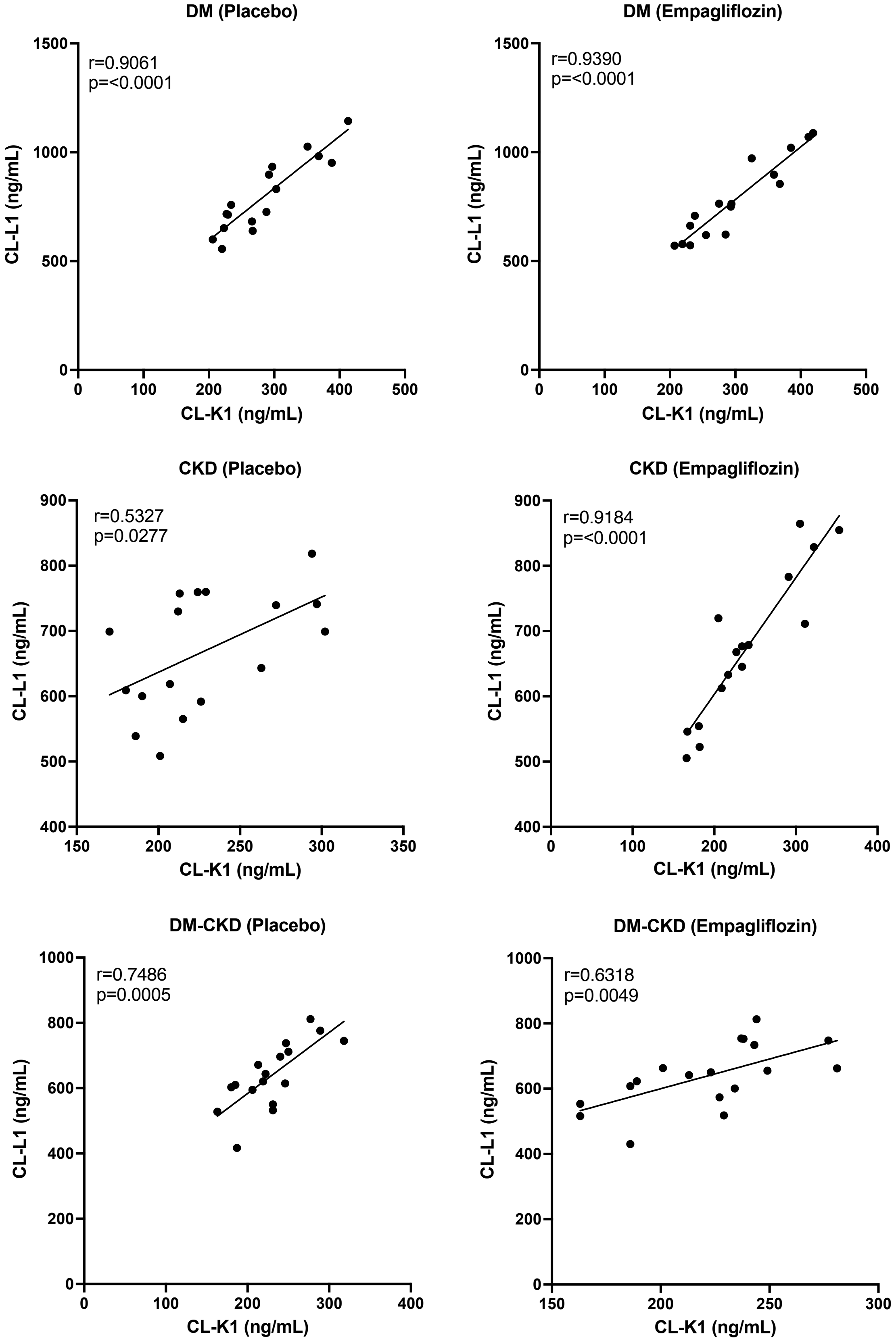

Figure S2: Correlation between plasma concentrations of the pattern recognition molecules collectin kidney 1 (CL-K1) and collectin liver 1 (CL-L1) after dapagliflozin and placebo treatment for DM (A and B), CKD (C and D), and DM-CKD (E and F). P<0.05 were considered statistically significant.

Supplementary Figure S3

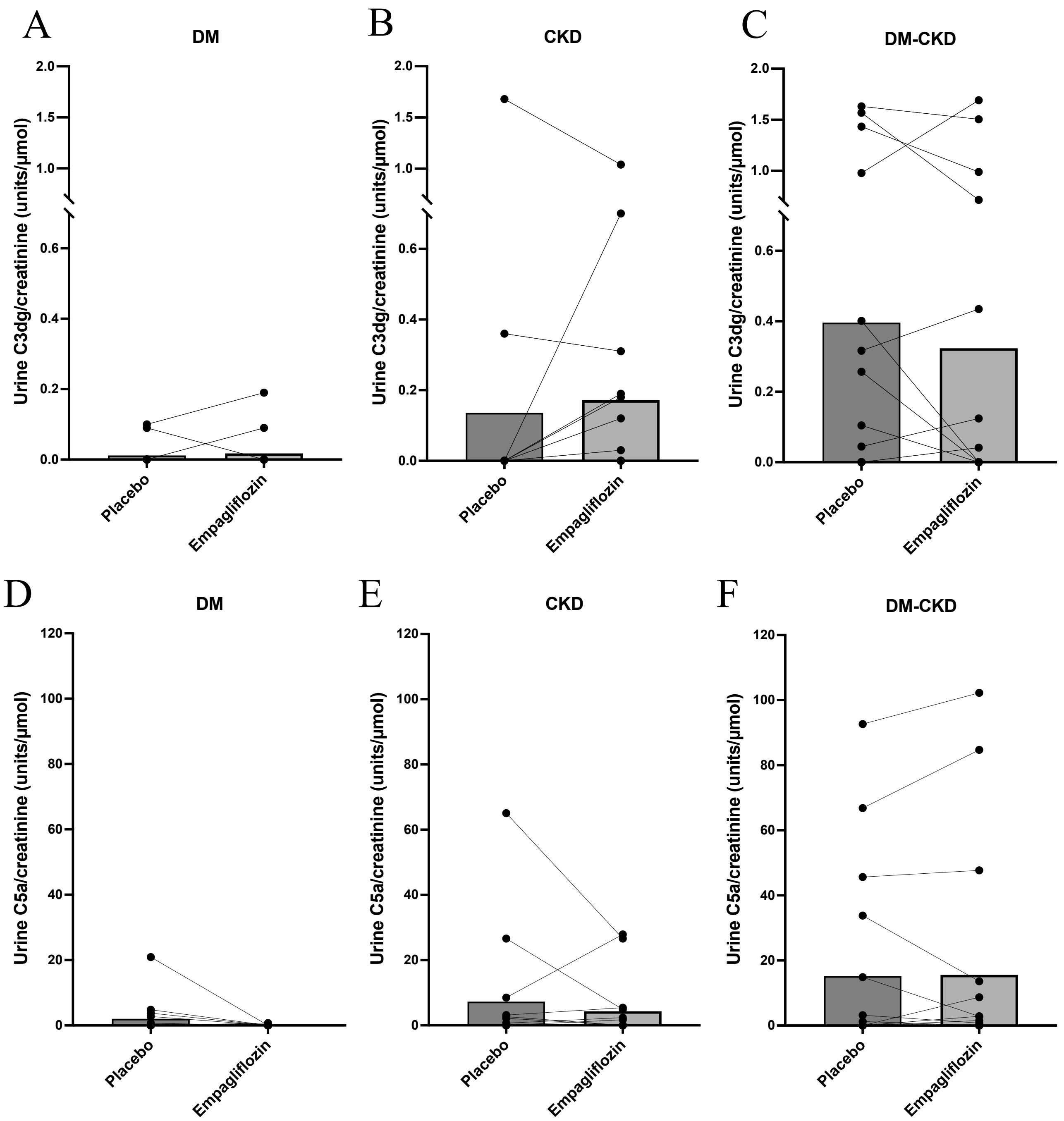

Figure S3: Spot urine complement activation product concentration shown as creatinine ratios for C3dg and C5a. The C3dga/creatinine ratio showed no significant differences between patients treated with placebo and empagliflozin in groups A) DM, B) CKD, and C) DM-CKD. No significant differences were observed in C5a levels between placebo and empagliflozin in D) DM, E) CKD, or F) DM-CKD. Both C3dg and C5a were normalized to urine creatinine. Results were interpreted as significant if $P < 0.05$.

Supplementary Table S1

Table S1: Concentration of lectin pathway-associated molecules in plasma from patients with DM, CKD, and DM-CKD in response to inhibition of SGLT2 by empagliflozin treatment for 4 weeks

	**DM (n=16)**		**CKD (n=16)**		**DM-CKD (n=17)**	
	Placebo	Empagliflozin	Placebo	Empagliflozin	Placebo	Empagliflozin
Collectins						
CL-K1 (ng/mL)	285.8 ± 64.7	299.8 ± 70.3[†]	224.0 ± 39.5	240.4 ± 58.9	229.6 ± 40.6	220.3 ± 35.1
CL-L1 (ng/mL)	800.5 ± 170.7	781.6 ± 181.1[†]	664.9 ± 92.3	675.2 ± 114.5	638.9 ± 101.6	632.0 ± 97.6
MBL (Units/mL)	23.32 ± 19.5	20.24 ± 15.7	22.0 ± 22.4	20.0 ± 20.3	15.5 ± 17.0	16.9 ± 17.2
Serine protease						
MASP-2 (ng/mL)	336.9 [255-391]	348.2 [236-406]	354.6 ± 147.0	365.1 ± 104.5	323.7 [270-402]	333.4 [282-411]

Data are presented as mean ± SD or median [IQR]. Abbreviations: DM, Diabetes mellitus. CKD, Chronic kidney disease. Significant difference between the groups: †

Supplementary Table S2

Table S2: Concentration of complement activation products in plasma from patients with DM, CKD, and DM-CKD in response to SGLT-2 inhibition by empagliflozin for 4 weeks

	**DM (n=16)**		**CKD (n=16)**		**DM-CKD (n=17)**	
	Placebo	Empagliflozin	Placebo	Empagliflozin	Placebo	Empagliflozin
C3a (ng/mL)	1638 [1384-2684]	1624 [1390-5110]	1325 [979-2579]	1387 [1241-1885]	2263 [1529-9451]	2632 [1683-3943][†]
C3dg (units/mL)	105.3 [77-125]	114 [84-160]	112.3 [84-141]	132.2 [87-186]	114.1 [91-139]	131.9 [98-157][*]
C5a (units/mL)	133.1 [95-191]	140.1 [111-239]	126.0 [89-201]	123.8 [90-151]	41 [33-60]	54 [40-68][†]
sC5b-9 (units/mL)	212.3 ± 77.6	205.4 ± 63.5	314.0 ± 186.1	276.4 ± 117.4	236.2 ± 81.8	232.3 ± 57.8

Data are presented as mean ± SD or median [IQR]. Abbreviations: DM, Diabetes mellitus. CKD, Chronic kidney disease. Significant difference intragroup between treatments: *P<0.05. Significant difference between the groups: †

Supplementary Table S3

Table S3: The difference between both evaluation periods in complement products in plasma and urine for placebo and empagliflozin treatment for DM

	**Placebo treatment**			**Empagliflozin treatment**		
Plasma	Period 1	Period 2	P-value	Period 1	Period 2	P-value
Collectins						
CL-K1 (ng/mL)	281.4 ± 72.5	290.1 ± 60.6	0.7973	294.0 ± 70.4	305.5 ± 74.5	0.7559
CL-L1 (ng/mL)	827.6 ± 187.1	773.4 ± 160.5	0.5442	778.0 ± 188.1	785.1 ± 186.7	0.9406
MBL (Units/mL)	22.1 ± 17.6	24.53 ± 22.3	0.8141	20.2 ± 15.1	20.2 ± 17.3	0.9994
Serine protease						
MASP-2 (ng/mL)	360.2 [242-400]	266.8 [255-376]	0.5054	325.6 [223-398]	351.6 [242-418]	0.9073
Complement activation products						
C3a (ng/mL)	1906 [1476-2684]	1574 [1354-8031]	0.2619	1624 [1442-8116]	2298 [1362-5110]	0.6454
C3dg (units/mL)	97.2 ± 24.4	105.7 ± 42.4	0.6313	121.5 ± 64.0	132.5 ± 44.8	0.6981
C5a (units/mL)	131.8 [93-241]	133.1 [101-191]	0.7984	182.3 [129-265]	120.1 [100-147]	0.1949
sC5b-9 (units/mL)	193.1 ± 81.0	231.6 ± 74.1	0.3387	229.9 ± 63.1	180.8 ± 57.2	0.1252
Urine						
Complement activation products						
C3a/Creatinine (ng/µmol)	3.2 [1.8-40]	9.2 [4.8-15]	0.2786	4.0 [2.4-6.1]	4.5 [1.5-27]	0.6236
C3dg/Creatinine (units/µmol)	0.0 [0.0-0.1]	0.0 [0.0-0.0]	0.1496	0.0 [0.0-0.0]	0.0 [0.0-0.1]	0.4667
C5a/Creatinine (units/µmol)	0.0 [0.0-2.8]	0.0 [0.0-2.2]	0.8564	0.0 [0.0-0.0]	0.0 [0.0-0.0]	>0.9999
sC5b-9/Creatinine (units/µmol)	0.0 [0.0-1.6]	0.0 [0.0-0.13]	0.6867	0.04 [0.0-0.2]	0.4 [0.0-0.4]	0.5888

Data are presented as mean ± SD or median [IQR]. Abbreviations: DM, Diabetes Mellitus

Supplementary Table S4

Table S4: The difference between both evaluation periods in complement products in plasma and urine for placebo and empagliflozin treatment for CKD

	**Placebo treatment**			**Empagliflozin treatment**		
Plasma	Period 1	Period 2	P-value	Period 1	Period 2	P-value
Collectins						
CL-K1 (ng/mL)	235.4 ± 42.7	220.3 ± 42.9	0.4764	229.1 ± 55.7	251.6 ± 63.5	0.4637
CL-L1 (ng/mL)	673.8 ± 88.9	664.4 ± 99.7	0.8390	669.2 ± 130.4	681.2 ± 105.0	0.8425
MBL (Units/mL)	18.4 ± 17.9	26.6 ± 25.9	0.4589	24.2 ± 23.5	15.8 ± 17.1	0.4339
Serine protease						
MASP-2 (ng/mL)	388.2 [251-523]	345.9 [216-467]	0.3593	360.3 [310-419]	334.6 [250-572]	>0.9999
Complement activation products						
C3a (ng/mL)	1178 [872-3931]	1789 [979-2579]	0.6058	1672 [1247-4957]	1350 [1229-1578]	0.1436
C3dg (units/mL)	83.7 [63-115]	130.0 [105-252]	0.0206*	164.5 [86-202]	114.1 [88-175]	0.6177
C5a (units/mL)	137.1 ± 50.0	180.0 ± 100.1	0.2727	168.6 ± 82.2	108.2 ± 24.7	0.0663
sC5b-9 (units/mL)	301.8 ± 208.0	320.7 ± 158.0	0.8377	273.2 ± 79.6	279.6 ± 152.3	0.9180
Urine						
Complement activation products						
C3a/Creatinine (ng/µmol)	11.4 ± 14.8	106.8 ± 159.8	0.0933	89.24 ± 114.9	11.5 ± 15.6	0.0787
C3dg/Creatinine (units/µmol)	0.0 [0.0-0.0]	0.0 [0.0-0.46]	0.0625	0.1 [0.0-0.6]	0.0 [0.0-0.02]	0.0928
C5a/Creatinine (units/µmol)	0.0 [0.0-2.4]	3.1 [0.0-15.6]	0.1320	2.4 [0.0-21.3]	0.0 [0.0-1.3]	0.0971
sC5b-9/Creatinine (units/µmol)	0.0 [0.0-1.6]	1.1 [0.2-9.3]	0.1100	0.3 [0.0-4.7]	0.1 [0.0-2.2]	0.4182

Data are presented as mean ± SD or median [IQR]. Abbreviations: CKD, Chronic kidney Disease. Significant difference: *P<0.05

Supplementary Table S5

Table S5: The difference between both evaluation periods in complement products in plasma and urine for placebo and empagliflozin treatment for DM-CKD

	**Placebo treatment**			**Empagliflozin treatment**		
Plasma	Period 1	Period 2	P-value	Period 1	Period 2	P-value
Collectins						
CL-K1 (ng/mL)	224.3 ± 44.1	235.6 ± 38.3	0.5839	226.8 [34.3]	215.8 [35.4]	0.5131
CL-L1 (ng/mL)	640.5 ± 68.9	637.2 ± 134.7	0.9484	630.8 ± 126.0	646.5 ± 69.1	0.7470
MBL (Units/mL)	18.5 [2.4-34]	3.7 [2.0-12]	0.2766	6.3 [1.8-15]	26.6 [1.8-39]	0.3213
Serine protease						
MASP-2 (ng/mL)	318.3 ± 67.9	403.7 ± 193.1	0.2318	412.9 ± 191.1	322.1 ± 56.3	0.1923
Complement activation products						
C3a (ng/mL)	2345 [1927-9917]	1997 [1399-8690]	0.4807	2071 [1576-3843]	2762 [2198-6745]	0.4234
C3dg (units/mL)	98.9 ± 28.4	137.6 ± 38.6	0.0314*	167.1 ± 76.7	112.6 ± 26.2	0.0626
C5a (units/mL)	46.7 ± 16.3	43.5 ± 13.3	0.6659	57.2 ± 18.1	53.2 ± 21.5	0.6904
sC5b-9 (units/mL)	189.9 [181-283]	213.3 [176-368]	0.8884	234.2 [202-316]	212.6 [177-236]	0.2359
Urine						
Complement activation products						
C3a/Creatinine (ng/µmol)	48.0 [4.4-285]	4.2 [2.0-196]	0.1672	3.7 [0.4-77]	9.9 [2.3-206]	0.1937
C3dg/Creatinine (units/µmol)	0.2 [0.0-1.3]	0.02 [0.0-0.3]	0.04461	0.0 [0.0-0.3]	0.0 [0.0-1.1]	0.6665
C5a/Creatinine (units/µmol)	1.2 [0.0-56]	0.0 [0.0-2.4]	0.1781	1.1 [0.0-7.2]	0.0 [0.0-66]	0.1405
sC5b-9/Creatinine (units/µmol)	1.0 [0.3-17]	1.0 [0.1-26]	0.7266	0.4 [0.3-9.2]	0.5 [0.0-19]	0.7420

Data are presented as mean ± SD or median [IQR]. Abbreviations: DM, Diabetes Mellitus. CKD, Chronic Kidney Disease.

Supplementary Table S6

Table S6: Correlation between urinary complement activation products and urine albumin

		Correlation to urinary albumin excretion			
Group	Treatment	C3a	C3dg	C5a	sC5b-9
DM	Placebo	0.4874 (0.0575)	0.1604 (0.5652)	0.3646 (0.1654)	0.7138 (0.0028**)
	Empagliflozin	0.5220 (0.0402*)	0.2402 (0.3208)	-0.1462 (0.9375)	0.4297 (0.0988)
CKD	Placebo	0.6222 (0.0116*)	0.4879 (0.0574)	0.5595 (0.0265*)	0.6974 (0.0037**)
	Empagliflozin	0.5651 (0.0245*)	0.3146 (0.2335)	0.2038 (0.4451)	0.6747 (0.0054**)
DM-CKD	Placebo	0.8351 (<0.0001****)	0.6187 (0.0098**)	0.5787 (0.0167*)	0.8045 (0.0002***)
	Empagliflozin	0.8650 (<0.0001****)	0.8018 (0.0002***)	0.7179 (0.0018**)	0.9367 (<0.0001****)

Data are presented as correlation coefficient r and (two-tailed p-value). Results were interpreted as significant if P <0.05, *P<0.05, **P<0.01, ***P<0.001, ****P<0.0001

Supplementary references:

S1. Nangaku M, Pippin J, and Couser WG. Complement membrane attack complex (C5b-9) mediates interstitial disease in experimental nephrotic syndrome. *J Am Soc Nephrol* 10: 2323-2331, 1999.

S2. Rangan GK, Pippin JW, Coombes JD, and Couser WG. C5b-9 does not mediate chronic tubulointerstitial disease in the absence of proteinuria. *Kidney Int* 67: 492-503, 2005.

S3. Rahmoune H, Thompson PW, Ward JM, Smith CD, Hong G, and Brown J. Glucose transporters in human renal proximal tubular cells isolated from the urine of patients with non-insulin-dependent diabetes. *Diabetes* 54: 3427-3434, 2005.

S4. Lu YP, Wu HW, Zhu T, Li XT, Zuo J, Hasan AA, Reichetzeder C, Delic D, Yard B, Klein T, Kramer BK, Zhang ZY, Wang XH, Yin LH, Dai Y, Zheng ZH, and Hocher B. Empagliflozin reduces kidney fibrosis and improves kidney function by alternative macrophage activation in rats with 5/6-nephrectomy. *Biomed Pharmacother* 156: 113947, 2022.

S5. Chen X, Hocher CF, Shen L, Kramer BK, and Hocher B. Reno- and cardioprotective molecular mechanisms of SGLT2 inhibitors beyond glycemic control: from bedside to bench. *Am J Physiol Cell Physiol* 325: C661-C681, 2023.

S6. Nielsen SF, Duus CL, Buus NH, Bech JN, and Mose FH. Empagliflozin in type 2 diabetes with and without CKD and non-diabetic CKD: Protocol for 3 randomized, double-blind, placebo controlled cross-over trials. *JMIR Res Protoc* 2024.

S7. Selman L, Henriksen ML, Brandt J, Palarasah Y, Waters A, Beales PL, Holmskov U, Jorgensen TJ, Nielsen C, Skjodt K, and Hansen S. An enzyme-linked immunosorbent assay (ELISA) for quantification of human collectin 11 (CL-11, CL-K1). *J Immunol Methods* 375: 182-188, 2012.

S8. Axelgaard E, Jensen L, Dyrlund TF, Nielsen HJ, Enghild JJ, Thiel S, and Jensenius JC. Investigations on collectin liver 1. *J Biol Chem* 288: 23407-23420, 2013.

S9. Palarasah Y, Nielsen C, Sprogoe U, Christensen ML, Lillevang S, Madsen HO, Bygum A, Koch C, Skjodt K, and Skjoedt MO. Novel assays to assess the functional capacity of the classical, the alternative and the lectin pathways of the complement system. *Clin Exp Immunol* 164: 388-395, 2011.

S10. Moller-Kristensen M, Jensenius JC, Jensen L, Thielens N, Rossi V, Arlaud G, and Thiel S. Levels of mannan-binding lectin-associated serine protease-2 in healthy individuals. *J Immunol Methods* 282: 159-167, 2003.

S11. Isaksson GL, Hinrichs GR, Andersen H, Bach ML, Weyer K, Zachar R, Henriksen JE, Madsen K, Lund IK, Mollet G, Bistrup C, Birn H, Jensen BL, and Palarasah Y. Amiloride Reduces

Urokinase/Plasminogen-Driven Intratubular Complement Activation in Glomerular Proteinuria. *J Am Soc Nephrol* 2024.

S12. Rasmussen KJ, Skjoedt MO, Vitved L, Skjoedt K, and Palarasah Y. A novel antihuman C3d monoclonal antibody with specificity to the C3d complement split product. *J Immunol Methods* 444: 51-55, 2017.

S13. Troldborg A, Halkjaer L, Pedersen H, Hansen A, Loft AG, Lindegaard H, Stengaard-Pedersen K, Graversen JH, Palarasah Y, and Thiel S. Complement activation in human autoimmune diseases and mouse models; employing a sandwich immunoassay specific for C3dg. *J Immunol Methods* 486: 112866, 2020.

S14. Wurzner R, Xu H, Franzke A, Schulze M, Peters JH, and Gotze O. Blood dendritic cells carry terminal complement complexes on their cell surface as detected by newly developed neoepitope-specific monoclonal antibodies. *Immunology* 74: 132-138, 1991.

S15. Isaksson GL, Nielsen MB, Hinrichs GR, Krogstrup NV, Zachar R, Stubmark H, Svenningsen P, Madsen K, Bistrup C, Jespersen B, Birn H, Palarasah Y, Jensen BL, and Group CS. Proteinuria is accompanied by intratubular complement activation and apical membrane deposition of C3dg and C5b-9 in kidney transplant recipients. *Am J Physiol Renal Physiol* 322: F150-F163, 2022.

S16. Rider MA, Hurwitz SN, and Meckes DG, Jr. ExtraPEG: A Polyethylene Glycol-Based Method for Enrichment of Extracellular Vesicles. *Sci Rep* 6: 23978, 2016.
